# Global high-risk clones and multidrug-resistant serotype strains of *Pseudomonas aeruginosa* from surgical site infections in low-resource settings

**DOI:** 10.1128/spectrum.00314-25

**Published:** 2025-10-30

**Authors:** Beverly Egyir, Rhodalyn Tagoe, Nicholas T. K. D. Dayie, Jeannette Bentum, Christian Owusu-Nyantakyi, Daniel Kwaku Baka, Blessing Kofi Adu Tabi, Appiah-Korang Labi, Noah Obeng-Nkrumah, Eric Behene, Salamatu Ibrahim, Felicia Owusu, Francis Adjei, Bright Agbodzi, Selassie Kumordjie, Julian Adinkrah, Awuiwenyuei Annankra, Isaac Yeboah, William Asiedu, Michael Adufutse, Philip Asare Annor Nyinaku, Edward Owusu Nyarko, Edward Asumanu, Harriet Manu, Naiki Attram, Robert D. Hontz, Anne Fox, Hugo Miranda Quijada, Terrel Sanders

**Affiliations:** 1 Bacteriology Department, Noguchi Memorial Institute for Medical Research, University of Ghana 58835, Accra, Ghana; 2 U.S. Naval Medical Research Unit EURAFCENT, Accra, Ghana; 3 Department of Medical Microbiology, University of Ghana Medical School 63533, Accra, Ghana; 4 Department of Medical Laboratory Sciences, School of Biomedical and Allied Health Sciences, University of Ghana 660620 , Accra, Ghana; 5 37 Military Hospital, Accra, Ghana; Hartford Hospital, Hartford, Connecticut, USA

**Keywords:** whole-genome sequencing, antimicrobial resistance, *Pseudomonas aeruginosa*, low-and middle-income countries, public health surveillance

## Abstract

Surgical site infections (SSIs) caused by *Pseudomonas aeruginosa* pose significant challenges in healthcare, particularly in low and middle-income countries (LMICs). Understanding the genomic characteristics and antimicrobial resistance profiles of *P. aeruginosa* isolates is crucial for informing appropriate treatment and guiding infection prevention and control strategies. *P. aeruginosa* isolates recovered from SSIs were analyzed using whole-genome sequencing and antimicrobial susceptibility testing. The isolates were identified using matrix assisted laser desorption ionization time of flight mass spectrometry (MALDI-ToF MS) system, and their antibiotic susceptibilities were determined using the Kirby–Bauer disk diffusion method and interpreted with Clinical and Laboratory Standards Institute guidelines. Whole-genome sequencing was performed using the Illumina MiSeq platform, and sequence data were analyzed using free online bioinformatics tools, including CARD-RGI, ResFinder, PAst, VFDB, and MLST. Between July 2018 and September 2023, 563 patients were enrolled in a surgical site infection study, which led to the recovery of 82 *P*. *aeruginosa* isolates. The isolates were resistant to ciprofloxacin (*n* = 23, 28.05%), gentamicin (*n* = 20, 24.39%), piperacillin-tazobactam (*n* = 13, 15.85%), cefepime (*n* = 12, 14.63%), ceftazidime (*n* = 12, 14.63%), meropenem (*n* = 10, 12.20%), and amikacin (*n* = 8, 9.76%). Multidrug resistance was noted in 10 isolates (12.20%), with three isolates resistant to all antibiotics tested (*n* = 3, 3.7%). Sequence data revealed international high-risk clones: ST308 and ST773 (*n* = 5 each, 6.10%), ST244 (*n* = 4, 4.88%), ST446, ST357, and ST654 (*n* = 2 each, 2.44%), and ST235 (*n* = 1, 1.41%). Newly identified sequence types ST4287, ST4288, ST4289, ST4521, ST4522, ST4523, and ST4524 were also detected. Serotype O11 (*n* = 28, 34.15%) was the most prevalent, followed by O5, O6, and O4. Key virulence genes, such as *algD*, *lasB*, *exoS*, *plcH*, *toxA*, and *plcN*, were also detected. The identification of both high-risk and novel clones, coupled with diverse resistance and virulence factors, underscores the need for continuous surveillance of circulating strains. Employing advanced technologies, such as whole-genome sequencing, is essential for guiding infection control and treatment strategies in Ghanaian hospital settings.

**IMPORTANCE:**

Surgical site infections (SSIs) caused by multidrug-resistant *Pseudomonas aeruginosa* are a major challenge to patient care, particularly in low-resource settings. This study reveals extensive genomic diversity, antimicrobial resistance, and virulence profiles among *P. aeruginosa* isolates from SSIs in Ghanaian hospitals. The detection of globally recognized high-risk clones ST235, ST308, and ST773, alongside several novel sequence types, suggests ongoing genomic evolution and the potential for regional dissemination. The presence of resistance to last-line antibiotics and key virulence factors highlights the therapeutic and public health challenges in low and middle-income countries. These findings underscore the urgent need for genomic surveillance, strengthened infection prevention and control, and targeted antimicrobial stewardship programs to inform treatment strategies and health policies across Ghana and the broader African region.

## INTRODUCTION

Surgical site infections (SSIs) are a major healthcare challenge, particularly in low and middle-income countries, contributing significantly to prolonged hospitalization, increased risk of infectious spread within hospital facilities, and increased healthcare expenses (1–3). With very limited resources in these settings, infection prevention and control (IPC) strategies are hampered and impose huge burdens on patients and healthcare systems (4). Among the myriad pathogens implicated in SSIs, *Pseudomonas aeruginosa* stands out due to its capacity to cause acute and chronic infections, both in and out of healthcare facilities (5, 6). Recognizing the threat posed by *P. aeruginosa*, the World Health Organization has classified it as a critical priority pathogen and grouped it in the ESKAPE pathogens, emphasizing the need for continuous monitoring and control. This pathogen cohort, consisting of *Enterococcus faecium*, *Staphylococcus aureus*, *Klebsiella pneumoniae*, *Acinetobacter baumannii*, *Pseudomonas aeruginosa*, and *Enterobacter* spp., is noted for its increasing multidrug resistance, emphasizing the urgent need for extensive surveillance, detection, and intervention (7, 8).

The high adaptability and resistance mechanisms in *P. aeruginosa* complicate infection management due to the growing resistance to antimicrobials and an array of virulence factors in its versatile genome (9, 10). Evidence of globally disseminated *P. aeruginosa* high-risk clones with enhanced virulence capacity, such as bacterial motility, biofilm formation, siderophore production, and spontaneous mutation rates has also been reported in many countries (11, 12). The rise of multidrug-resistant (MDR), extensively drug-resistant (XDR), pan-drug-resistant (PDR), and carbapenem-resistant *Pseudomonas aeruginosa* strains has raised global concerns, with outbreak reports in critical care units, burn units, and surgical wards (13–15). Despite its centrality as a healthcare-associated pathogen, research studies on the prevalence, genetic diversity, and population structure of *P. aeruginosa* in Africa are limited (16). This study aimed to address this knowledge gap by investigating the antimicrobial resistance profiles and genomic characteristics of *P. aeruginosa* recovered from surgical site infections in two major Ghanaian hospitals.

## MATERIALS AND METHODS

### Study design, site, and sampling procedures

This cross-sectional study was conducted in the surgical departments of two tertiary health facilities: Korle Bu Teaching Hospital (KBTH) and 37 Military Hospital (37MH), in Accra, between July 2018 and September 2023, following ethics committee approvals from the respective institutions. Each study participant was enrolled in the study once informed consent was obtained.

A sterile cotton-tipped applicator or syringe, was used to aseptically collect samples (wound swab, fluid, or aspirate), which were placed into Amies transport medium and transported to the Noguchi Memorial Institute for Medical Research (NMIMR) for phenotypic and genomic analysis.

### Bacterial culture and identification

The samples were plated on blood agar (Oxoid, USA) and MacConkey agar (BD Difco, USA) and incubated aerobically at 37°C. *P. aeruginosa* isolates were identified through the analysis of colonial morphologies on the respective media, Gram staining (Becton, Dickinson and Company, USA), oxidase testing (Becton, Dickinson and Company, USA), and catalase testing (Becton, Dickinson and Company, Mexico) and confirmed using matrix assisted laser desorption ionization time of flight mass spectrometry identification system (Bruker, USA).

### Antimicrobial susceptibility testing

Antimicrobial susceptibility testing was performed using the Kirby–Bauer disk diffusion method, and the results were interpreted according to the 2025 Clinical and Laboratory Standards Institute guidelines. The antibiotics tested against the bacteria were ceftazidime (30 µg), piperacillin-tazobactam (100/10 µg), meropenem (10 µg), cefepime (30 µg), and ciprofloxacin (5 µg). Multidrug resistance (MDR) was defined as resistance to at least one agent in at least three antibiotic classes.

### DNA extraction, whole-genome sequencing, and sequence analysis

Genomic DNA extraction was carried out using the QIAamp DNA Mini Kit (Qiagen, Germany). The concentrations of the DNA extracts were determined using the Qubit 2.0 fluorometer (Thermo Fisher Scientific, USA), and the genomic libraries were prepared using the Illumina DNA Prep Kit (Illumina Inc., San Diego, CA, USA) per the manufacturer’s instructions. The libraries were then quantified using a 2100 Agilent bioanalyzer system and sequenced on the Illumina MiSeq platform (Illumina Inc., San Diego, CA, USA), which generated 2 × 300 base paired end reads. The sequencing reads were trimmed using Trimmomatic v.0.39 (17) and quality-checked using FastQC v.0.12.0 (18). Trimmed reads were assembled using Unicycler v.0.5.0 (19).

The assembled sequence reads were analyzed via the Centre for Genomic Epidemiology’s KmerFinder v.4.1 (https://cge.food.dtu.dk/services/KmerFinder/) to determine species identity, PAst v.1.0 (https://cge.food.dtu.dk/services/PAst/) to determine the serotypes of the *P. aeruginosa* strains, ResFinder v.4.1 (https://cge.food.dtu.dk/services/ResFinder/) and Comprehensive Antibiotic Resistance Database (CARD) v.3.0.9 (https://card.mcmaster.ca/) to determine antibiotic resistance determinants, and Virulence Finder Database (VFDB) (http://www.mgc.ac.cn/cgi-bin/VFs/v5/main.cgi/) to determine virulence determinants in the *P. aeruginosa* strains.

A whole-genome single-nucleotide polymorphism (SNP) phylogenetic tree was constructed using the CSI Phylogeny tool with the default settings (https://cge.food.dtu.dk/services/CSIPhylogeny/). Bactinspector v.0.1.3 (https://gitlab.com/antunderwood/bactinspector/) was used to select the best reference for the genomes. The relatedness among the isolates and their genomic traits was presented graphically using interactive Tree of Life (iTOL) v.5.0 (20). The sequence data generated have been submitted and deposited in the NCBI database under the BioProject number PRJNA1015653.

### Data analysis

Demographic data and laboratory results were entered into Microsoft Excel version 2019 for statistical analysis. The data were analyzed using descriptive statistics and presented using frequencies, percentages, and bar graphs.

## RESULTS

### Antimicrobial resistance of *P. aeruginosa* isolates

Among the 563 samples, 82 *P*. *aeruginosa* isolates (14.57%) were recovered, with 64 (78.05%, 64/82) originating from KBTH and the remaining 18 (21.95%, 18/82) from 37MH. Resistance to ciprofloxacin (*n* = 22, 26.83%), piperacillin-tazobactam (*n* = 11, 13.42%), cefepime (*n* = 11, 13.42%), ceftazidime (*n* = 9, 10.98%), and meropenem (*n* = 10, 12.20%) was observed among the isolates (Table 1).

**TABLE 1:** Antibiotic resistance patterns of *P. aeruginosa* isolates at 37MH and KBTH

Hospital	Category	Meropenem (*n*, %)	Ciprofloxacin (*n*, %)	Ceftazidime (*n*, %)	Cefepime (*n*, %)	Piperacillin-tazobactam (*n*, %)
KBTH 37MH Total	Resistant	7 (10.94) 3 (16.67) 10 (12.20)	15 (23.44) 7 (38.89) 22 (26.83)	6 (9.38) 3 (16.67) 9 (10.98)	7 (10.94) 4 (22.2) 11 (13.42)	8 (12.5) 3 (16.67) 11 (13.42)
KBTH 37MH Total	Intermediate	0 0 0	1 (15.63) 0 1 (12.20)	3 (4.69) 0 3 (3.66)	1 (15.63) 0 1 (12.20)	3 (4.69) 0 3 (3.66)
KBTH 37MH Total	Susceptible	57 (89.06) 15 (83.33) 72 (87.81)	48 (75) 11 (61.11) 59 (71.95)	55 (85.94) 15 (83.33) 70 (85.37)	56 (87.5) 14 (77.78) 70 (85.37)	53 (82.81) 15 (83.33) 68 (82.93)

**Fig 1:**
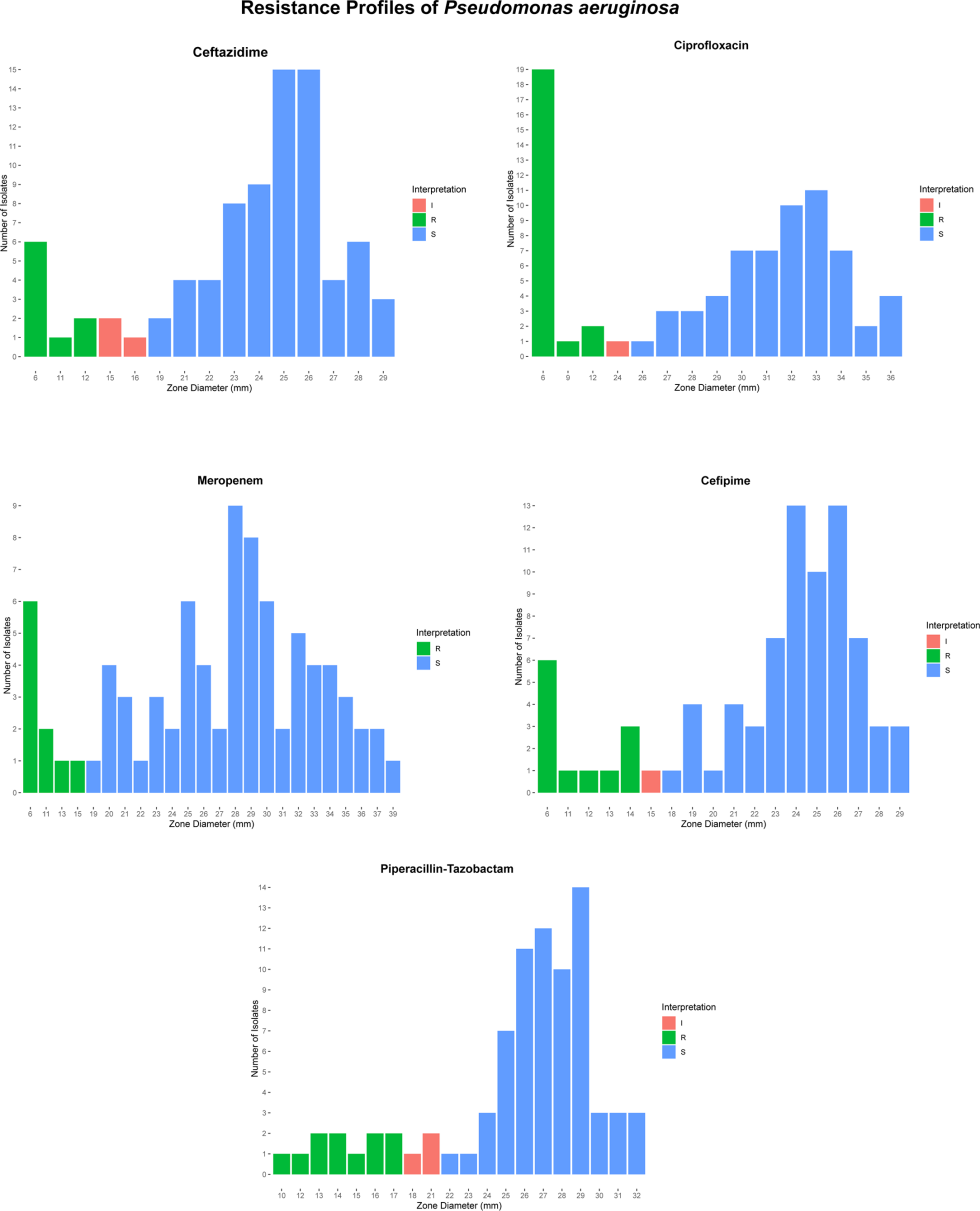
Resistance profiles of *P. aeruginosa* isolates. Zone diameter breakpoints were as follows: for ceftazidime and cefepime: resistant ≤14, intermediate 15–17, susceptible ≥18; for ciprofloxacin: resistant ≤18, intermediate 19–24, susceptible ≥25, for meropenem: resistant ≤15, intermediate 16–18, susceptible ≥ 19; for piperacillin-tazobactam: resistant ≤17, intermediate 18–21, susceptible ≥22;

Twenty-two (26.83%) isolates were resistant to at least one antibiotic class, and 10 (12.20%) were multidrug-resistant. Additionally, three isolates (3.7%) were resistant to all seven antibiotics tested. The resistance patterns of the isolates are depicted in Fig. 1.

### Genomic characteristics of *Pseudomonas aeruginosa*

Diverse sequence types were found among the isolates, seven of which were novel. The sequence types (STs) include ST308 and ST773 (*n* = 5 each), ST244 (*n* = 4), ST1684 (*n* = 3), ST1682, ST2483, ST254, ST261, ST277, ST357, ST381, ST3959, ST4289, ST446, ST4524, ST654, and ST897 (*n* = 2 each) and 33 singletons. The novel sequence types are ST4287, ST4288, ST4289, ST4521, ST4522, ST4523, and ST4524. The allelic profiles of the seven housekeeping genes in the novel sequence types are provided in Table 2. The predominant O-antigen serotypes among the isolates were O11 (*n* = 28, 34.15%), O5 (*n* = 17, 20.73%), O6 (*n* = 6, 7.31%), and O4 (*n* = 5, 6.10%).

**TABLE 2:** Allelic profiles of novel STs^*a*^

ID	Allelic profiles of novel STs								SLV/DLV
	ST	Housekeeping genes							
		*acsA*	*aroE*	*guaA*	*mutL*	*nuoD*	*ppsA*	*trpE*	
KBU-M-012	ST4288	38	11	3	13	**176** ^*b*^	2	4	SLV
KBU-045	ST4287	2	4	5	3	1	6	**357**	SLV
KBU-M-030	ST4289	**301**	30	61	26	30	59	15	SLV
TMH-011	ST4289	**301**	30	61	26	30	59	15	SLV
KBU-096	ST4521	**17**	5	12	3	14	4	**374**	DLV
KBU-103	ST4522	**320**	5	12	11	4	4	20	SLV
KBU-172	**ST4523**	111	30	64	26	28	24	143	
KBU-145	ST4524	11	**404**	26	3	4	4	14	SLV
KBU-260	ST4524	11	**404**	26	3	4	4	1	SLV

^*a*^ ST, sequence type; SLV, single locus variation; DLV, double locus variation.

^*b*^ Boldface indicates the new allele assignments in the newly assigned *P. aeruginosa* sequence types.

**Fig 2:**
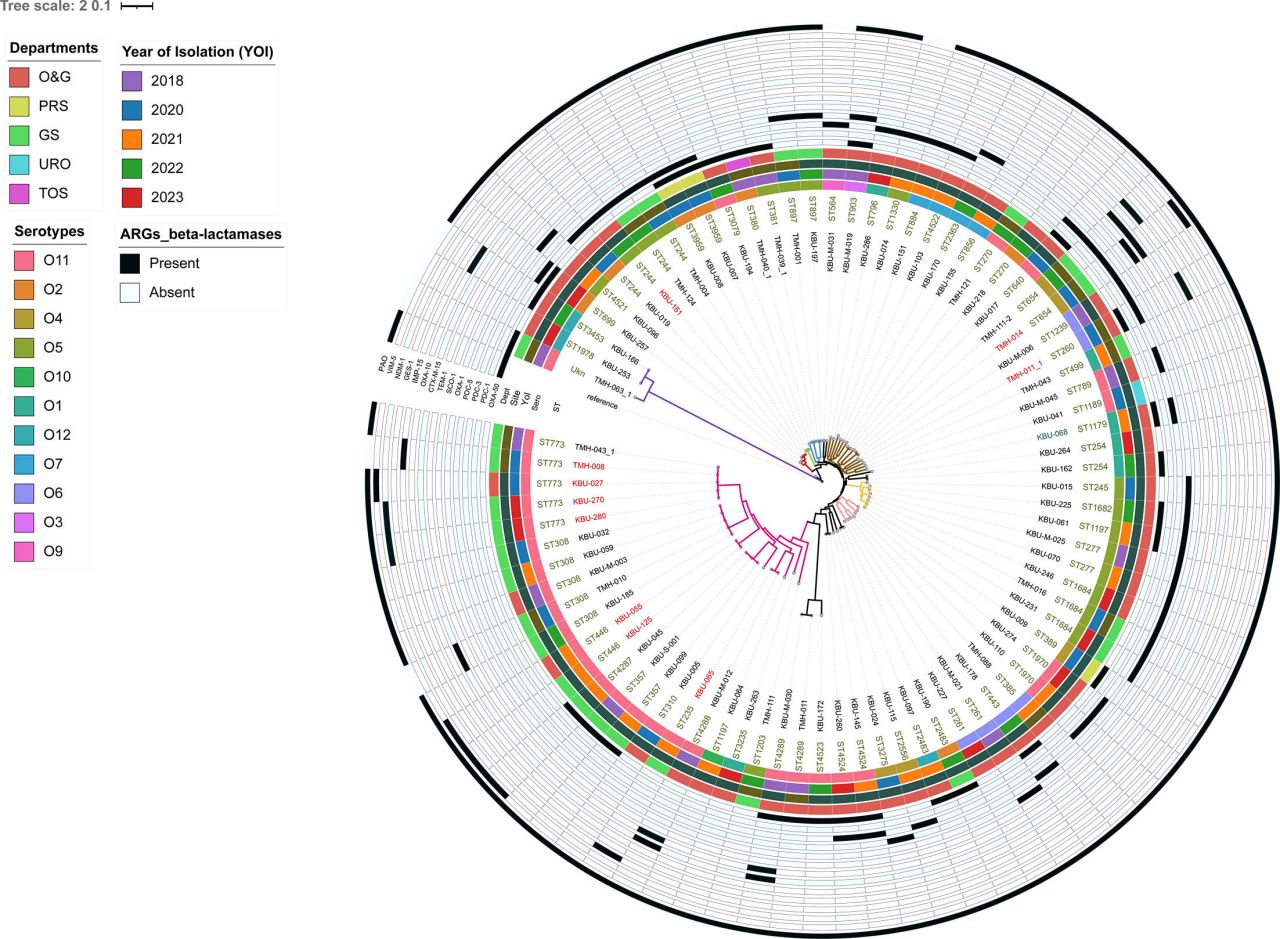
Core maximum-likelihood phylogeny of the 82 *P. aeruginosa* isolates. The phylogenetic tree was constructed based on single-nucleotide polymorphisms aligned to the reference strain, NC_002516.2. The phylogeny was inferred using CSI Phylogeny and annotated using iTOL with sequence types, serotype, year of isolation, department, and resistance genes as detected by the CARD and ResFinder. From the phylogenetic tree, some STS are observed to cluster, indicative of close-relatedness of strains. Annotated beta-lactamase resistance genes (inner to outer ring) include *bla*_*OXA-50*_, *bla*_*PDC-1*_*, bla*_*PDC-3*_*, bla*_*PDC-5*_*, bla*_*OXA-1*_*, bla*_*SCO-1*_*, bla*_*TEM-1*_*, bla*_*CTX-M-15*_*, bla*_*OXA-10*_*, bla*_*IMP-15*_*, bla*_*GES-1*_*, bla*_*NDM-1*_*, bla*_*VIM-5*_*,* and *bla*_*PAO*._

**Fig 3:**
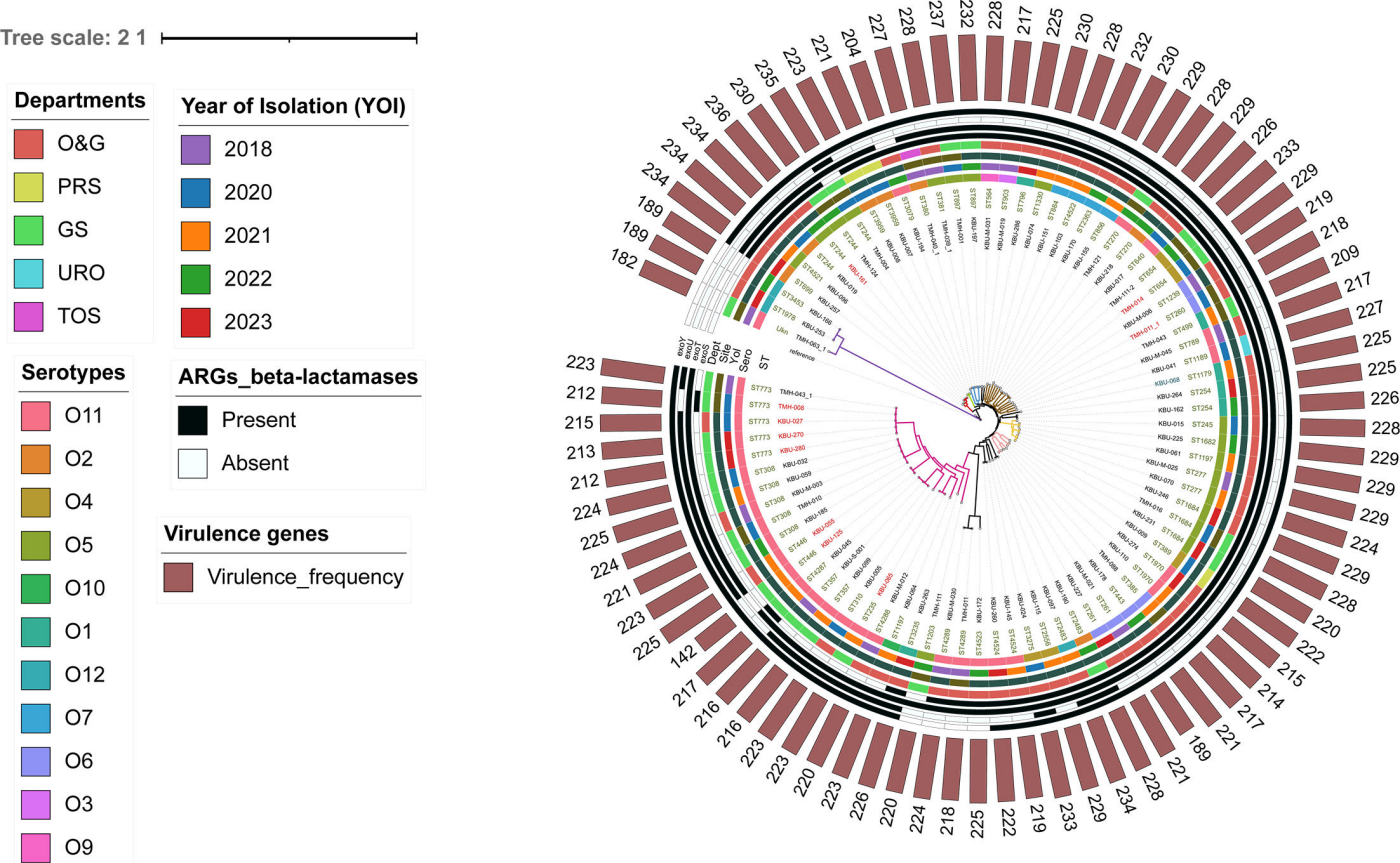
Presence or absence representation (as indicated by VFDB) of variably present virulence genes among the *Pseudomonas aeruginosa* isolate collection mapped on the maximum-likelihood phylogenetic tree. The phylogenetic tree was constructed based on single-nucleotide polymorphisms aligned to the reference strain, NC_002516.2. The phylogeny was inferred using CSI Phylogeny and annotated using iTOL, which included (inner to outer ring) sequence types, serotypes, year of isolation, department, and virulence genes. Annotated Type III secretion system/effector genes include *exoS*, *exoT*, *exoU*, and *exoY*. The number of virulene genes harbored by each isolate is reported on the outermost ring as a bar plot.

Pairwise SNP analysis revealed SNP differences (<10) between some *P. aeruginosa* isolates, suggesting recent common ancestry and potential transmission in some hospital departments. Specifically for ST308, a SNP difference of six was observed between TMH-008 and TMH-043_1, while KBU-032 and KBU-059 differed by three SNPs. Similarly, KBU-M-003 and KBU-059 (both ST308) differed by seven to eight SNPs. Within ST773, KBU-270 and KBU-280 showed a difference of one SNP, indicating clonal relatedness. Closely related isolates were linked to specific hospital departments. For example, ST308 was identified among isolates from the General Surgery department of Korle Bu Teaching Hospital (KBTH) (n=3) and from the Surgical Outpatient Department (OPD) of 37 Military Hospital (37MH) (Fig. 2 and 3).

A maximum-likelihood tree, which is based on SNP differences, was generated to determine phylogenetic relatedness among the isolates, as shown in Fig. 1.

### Antibiograms of international high-risk clones

Table 3 summarizes the 24 isolates, year of isolation, hospital, ward/department, serotype, sequence type, antibiograms, resistance gene content (carbapenemases and extended-spectrum beta-lactamases [ESBLs]), and Type (III) secretion system (T3SS) pathotypes of high-risk clones.

**TABLE 3:** Characteristics of *P. aeruginosa* international high-risk clones^*a*^

Sample ID	YoI	Hosp	Dept	ST	Ser	Surg	Antibiogram	Carbapenemases/ESBLs	T3SS pathotype
TMH-011_1	2018	37MH	O&G	260	O11	XLAP	MEM+CIP+FEP+PTZ	*bla_OXA-10_*	*exoU−/exoS+*
37MH-043	2018	37MH	GS	773	O11	XLAP	CIP	*–*	*exoU+/exoS−*
TMH-004	2020	37MH	GS	244	O5	APPY	–	*–*	*exoU+/exoS−*
TMH-008	2020	37MH	GS	773	O11	APPY	MEM+CIP+CAZ+FEP+PTZ	*bla_IMP-15_*	*exoU−/exoS+*
TMH-010	2020	37MH	O&G	308	O11	CSEC	CIP+CAZ+FEP	*bla_CTX-M-15_*	*exoU+/exoS−*
TMH-014	2020	37MH	GS	654	O4	XLAP	MEM+CIP+FEP+PTZ	*bla_SCO-1_, bla_TEM-1_, bla_VIM-5_*	*exoU−/exoS+*
TMH-111 (2)	2022	37MH	GS	654	O4	RECT	CIP+PTZ	*bla_TEM-1_, bla_OXA-10_*	*exoU−/exoS+*
TMH-124	2022	37MH	GS	244	O5	HPRO	–	*–*	*exoU−/exoS+*
KBU-M-003	2018	KBTH	O&G	308	O11	HYST	CIP	*–*	*exoU+/exoS−*
KBU-M-025	2018	KBTH	O&G	277	O5	CSEC	–	*–*	*exoU−/exoS+*
KBU-S-001	2018	KBTH	GS	357	O11	IMPL	CIP+FEP+PTZ	*–*	*exoU+/exoS−*
KBU-019	2020	KBTH	O&G	244	O2	HYST	CAZ+FEP+PTZ	*–*	*exoU−/exoS+*
KBU-027	2020	KBTH	O&G	773	O11	XLAP	MEM+CIP+CAZ+FEP+PTZ	*bla_VIM-5_*	*exoU+/exoS−*
KBU-032	2020	KBTH	GS	308	O11	XLAP	CIP	***–***	*exoU+/exoS−*
KBU-055	2021	KBTH	O&G	446	O11	XLAP	MEM+CIP+CAZ+FEP+PTZ	*bla_VIM-5_*	*exoU+/exoS−*
KBU-059	2021	KBTH	GS	308	O11	XLAP+APPY	CIP	*–*	*exoU+/exoS−*
KBU-065	2021	KBTH	GS	235	O11	BILI	MEM+CIP+CAZ+FEP+PTZ	*bla_GES-1_*	*exoU+/exoS−*
KBU-070	2021	KBTH	O&G	277	O5	HYST	–	*–*	*exoU−/exoS+*
KBU-099	2021	KBTH	GS	357	O5	URET	–	*–*	*exoU+/exoS−*
KBU-125	2021	KBTH	GS	446	O11	XLAP+COLO	MEM+CIP+CAZ+PTZ	*bla_VIM-5_*	*exoU−/exoS−*
KBU-161	2022	KBTH	O&G	244	O5	CSEC	MEM	*–*	*exoU−/exoS+*
KBU-185	2022	KBTH	GS	308	O11	XLAP	CIP	*–*	*exoU+/exoS−*
KBU-270	2023	KBTH	GS	773	O11	FIST	MEM+CIP+CAZ+FEP+PTZ	*bla_NDM-1_*	*exoU+/exoS−*
KBU-280	2023	KBTH	GS	773	O11	RECT	MEM+CIP+CAZ+FEP+PTZ	*bla_NDM-1_*	*exoU+/exoS−*

^*a*^ MEM, meropenem; YoI, year of isolation; Hosp, hospital; Dept, department; ST, sequence type; Surg, surgery; T3SS, type III secretion system; ESBLs, extended spectrum beta-lactamases; KBTH, Korle Bu Teaching Hospital; 37MH, 37 Military Hospital; O&G, obstetrics and gynecology; GS, general surgery; XLAP, exploratory laparotomy; CSEC, cesarian section; APPY, appendix surgery; BILI, bile duct, liver, or pancreatic surgery; FIST, fistulectomy; HYST, abdominal hysterectomy; COLO, colon surgery; HPRO, hip prosthesis; RECT, rectal surgery; URET, urethroplasty; IMPL, implant; FEP, cefepime; CAZ, ceftazidime; PTZ, piperacillin-tazobactam; CIP, ciprofloxacin; –, no resistance or gene.

### Resistance gene distribution

Using ResFinder v.4.1 (21) and Comprehensive Antibiotic Resistance Database-Resistance Gene Identifier (CARD-RGI) (22), genomic analysis revealed a variable distribution of resistance genes (*n* = 144). These resistance determinants encode resistance to 16 different antimicrobial classes and include aminoglycoside-modifying enzymes, cephalosporinases, carbapenemases, disinfectants, and heavy metal resistance determinants. An array of multidrug efflux systems was also identified.

Beta-lactam resistance determinants were the most diverse, with 46 uniquely occurring genes. Among these genes, 17 *Pseudomonas*-derived cephalosporinases (PDCs), 19 oxacillinases, 4 extended-spectrum beta-lactamases, and 4 carbapenemase genes were observed in the collection. Acquired resistance determinants from various bacterial species (e.g., *Haemophilus influenzae* PBP3 (D350N, S357N), *Salmonella enterica gyrA* (S83F), *Escherichia coli parC*, *Staphylococcus intermedius* chloramphenicol acetyltransferase, *E. coli emrE*) with action against different antimicrobial classes, as well as a host of efflux systems, were observed. Chloramphenicol (*P. aeruginosa catB7*), glycopeptide (*vanW* in *vanG* cluster), peptide (*cprR*, *cprS*, *arnA*, *bcr-1*), fosfomycin (*fosA*), and disinfectant (*triA*, *triB*, *triC*) resistance determinants were the dominant genes in more than 90% of the sequenced isolates. Table 4 summarizes the horizontally acquired beta-lactamases in the *P. aeruginosa* isolates.

**TABLE 4:** Horizontally acquired beta-lactamases in *P. aeruginosa* isolates^*a*^

Ambler class	Beta-lactamase type	Enzyme	Clones/ST
Class A	*bla_CTX-M_*	*bla_CTX-M-15_*	ST261 (1) **ST 308 (1),** ST4289 (1)
	*bla_TEM_*	*bla_TEM-1B_*	ST4288 (1), ST4289 (1), **ST654 (2)**
	*bla_SCO_*	*bla_SCO-1_*	ST4288 (1), **ST654 (1)**
	*bla_GES_*	*bla_GES-1_*	**ST235 (1)**
Class B	*bla_IMP_*	*bla_IMP-15_*	**ST773 (1)**
	*bla_NDM_*	*bla_NDM-1_*	**ST773 (2)**
	*bla_VIM_*	*bla_VIM-5_*	ST4287 (1), **ST773 (1), ST654 (1), ST446 (2)**
Class D	*bla_OXA_*	*bla_OXA-10_*	**ST260 (1),** ST3453 (1), **ST654 (1), ST773 (1)**
		*bla_OXA-21_*	**ST773 (1)**

^*a*^ The bold values represent the frequencies of horizontally-acquired beta-lactamases in widely-disseminated high-risk clones within the collection.

Quaternary ammonium compounds (QACs) and heavy metal resistance genes (*qacG*, *qacJ*, *qacL*, and *qacEδ1*) were sparsely distributed (*n* ≤ 20%) among the isolates. Determinants encoding resistance to peptides (*eptA*, *ugd*, *bacA*, *pmrF*, *yojI*, and *arnT*) (*n* ≤ 30.5%), nitroimidazoles (*msbA*) (*n* ≤ 1.2%), and aminocoumarins (e.g., novobiocin) (*mdtA*, *mdtB*, and *mdtC*) (*n* ≤ 1.2%) were also detected.

Of note, each *P. aeruginosa* isolate bore a PDC beta-lactamase gene, with *bla*_PDC-3_ being the most dominant (*n* = 17, 20.73%), followed by *bla*_*PDC-5*_ (*n* = 15, 18.29%), and known for their ESBL activity. Close associations of various PDCs to specific STs were observed. Among the widely disseminated clones, all five strains of ST773 and ST308 bore *bla*_*PDC-16*_ and *bla*_*PDC-19a*_, respectively. Similarly, the four ST244 strains carried *bla_PDC-1_*, while ST654 and ST277 (*n* = 2 each) harbored *bla*_*PDC-3*_ and *bla*_*PDC-5*_, respectively. Besides high-risk clones, ST3959 bore *bla*_*PDC-1*_ while ST1684 (*n* = 3) was with *bla*_*PDC-5*_, and the newly assigned ST4524 bore *bla*_*PDC-3*_. Table 5 summarizes the distribution of the PDCs variants among the *Pseudomonas aeruginosa* collection. The bold values represent observed frequencies for widely disseminated high-risk clones in the collection.

**TABLE 5:** PDCs variants among selected *Pseudomonas aeruginosa* strains^*a*^

PDC variant	Frequency	Sequence types
*bla*_*PDC-1*_	**7**	ST3959 (*n* = 2)**, ST244 (*n* = 4),** ST699
*bla*_*PDC-3*_	17	ST640, ST1179, ST1330, ST4522, ST1970, ST2556, ST4524 (*n* = 2), ST884, ST1970, ST796, ST1239, ST564, ST789, ST260, **ST654 (*n* = 2)**
*bla*_*PDC-5*_	15	ST249, ST3275, ST1682, **ST277 (*n* = 2)**, ST2363, ST443, ST897, ST1682, ST1684 (*n* = 3), ST903, ST897
*bla*_*PDC-16*_	7	**ST773 (*n* = 5), ST446 (*n* = 2)**
*bla*_*PDC-19a*_	6	**ST308 (*n* = 5),** ST1203
*bla*_*PDC-8*_	5	ST254 (*n* = 2), ST270 (*n* = 2), ST3079
*bla*_*PDC-11*_	3	**ST357 (*n* = 2),** ST4287
*bla*_*PDC-66*_	3	ST4289 (*n* = 2), ST4523
*bla*_*PDC-67*_	3	ST261 (*n* = 2), ST385
*bla*_*PDC-35*_	2	**ST235,** ST4288

^*a*^ The bold values represent observed frequencies for widely disseminated high-risk clones in the collection.

Several amino acid/gene mutations encoding resistance to several antimicrobial classes among the isolates were observed. Table 6 summarizes the mutational changes observed. Remarkably, one of our ST261 strains exhibited a large acquired resistome and multiple acquired point mutations in various resistance determinants. These traits originated from other bacterial species, including *E. coli cyaA* (*S353T*), *E. coli parC* (*S80I*), *H. influenzae PBP3* (*D87N*, *S83L*), *E. coli glpT* (*E448K*), *nalC* (*G71E*), and *basR* (*L71R*), among others.

**TABLE 6:** Resistance point mutations occurring in the resistomes of *P. aeruginosa* strains^*a*^

Gene	Antimicrobial	Mutation	Frequency
*nalC*	Peptide, sulfonamide, cephamycin, macrolide, monobactam, penam, tetracycline, fluoroquinolone, cephalosporin, carbapenem, phenicol, aminocoumarin	A186T	9
		G71E	66
		S209R	48
*basR*	Peptide	L71R	50
*P. aeruginosa gyrA*	Fluoroquinolone	T83I	17
*mexS*	Fluoroquinolone, phenicol, diaminopyrimidine	V73A	2
*E. coli parC*	Fluoroquinolone	S80I	1
*H. influenzae PBP3*	Penam, cephalosporin, cephamycin	D87N	1
		S83L	1
*E. coli glpT*	Fosfomycin	E448K	1
*E. coli acrAB-tolC* with *marR* mutation	Ciprofloxacin, tetracycline	Y137H	1
		G103S	1
*E. coli acrAB-tolC* with *acrR* mutation	Ciprofloxacin, tetracycline, ceftazidime		1
*E. coli cyaA*	Fosfomycin	S353T	1
*K. pneumoniae ramR*	Tetracycline, rifamycin, phenicol, penam, cephalosporin, disinfecting agents and antiseptics, glycylcycline, fluoroquinolone	A19V	1

^*a*^ A, alanine; D, aspartic acid; E, glutamic acid; F, phenylalanine; G, glycine; H, histidine; I, isoleucine; K, lysine; L, leucine; N, asparagine; Q, glutamine; R, arginine; S, serine; T, threonine; V, valine; Y, tyrosine.

### Virulence gene distribution

Virulome analysis revealed over 215 genes encoding determinants for adherence, antimicrobial activity, antiphagocytosis, enzymes, toxins, quorum sensing, secretion systems, iron uptake, biofilm formation, and stress adaptation, which are graphically represented in Fig. 2. Additional virulence determinants from other bacterial species, such as *Mycobacterium*, *E. coli*, *Haemophilus*, *Shigella*, *Neisseria*, *Klebsiella*, *Salmonella*, *Shigella*, *Bacillus*, and *Vibrio*, were found among the *P. aeruginosa* isolates. They include determinants for capsular polysaccharides, type 1 fimbriae, copper exporters, catalase-peroxidase, magnesium and manganese uptake, efflux pumps, biofilm and cell surface adherence components, iron uptake systems, hemolysins, and cytolysins, among others.

Among the virulence determinants, *algA*, *pvdH*, *lasI*, and *rhlI* were the most common (*n* = 80), followed closely by *pilT*, *pvdE*, and *rhlR* (*n* = 79), and several genes rarely occurred in the collection (*msbB2*, *wzc*, *rmlB*, *acrA*, *hlyA*, *hlyE*, *chuA*, *shuV*, *katG*, *mntB*, *mgtB*, *mgtC*, *katG*, *adeG*, *traT*, etc.).

### Type III secretion system (T3SS) pathotypes

The Type III secretion system effectors *exoS*, *exoT*, *exoU*, and *exoY* were found among the *P. aeruginosa* isolates. The distribution of T3SS pathotypes among the isolates was as follows: *exoU*−/*exoS*+ (55 isolates), *exoU*+/*exoS*− (18 isolates), *exoU*−/*exoS*− (5 isolates), and *exoU*+/*exoS*+ (4 isolates). Interestingly, the four isolates that co-occurred with mutually exclusive T3SS genes (*exoS* and *exoU*) presented no resistance to the antibiotics tested. The *exoU*+/*exoS*− pathotype was the most dominant (*n* = 14) among the high-risk clones in the collection, followed by *exoU*−/*exoS*+ (*n* = 9) and *exoU*−/*exoS*− (*n* = 1).

Among the five strains with the *exoU*−/*exoS*− pathotype, one harbored only the *exoY* effector gene (ST446), another harbored only the *exoT* effector gene (ST1203), and three harbored neither of the T3SS effectors (ST1978, ST3453, and unknown STs). However, exolysins (*exlA*, *exlB*), an acquired efflux system for biofilm formation (*Acinetobacter adeG*), and secretion systems from *Vibrio* (*epsE*, *epsG*) were present in their genomes.

## DISCUSSION

Antimicrobial resistance remains a “silent pandemic” within healthcare settings. This study provides insights into antimicrobial resistance profiles and whole-genome characteristics of *P. aeruginosa* isolates from SSIs. The findings reveal a concerning level of resistance to routinely used antipseudomonal agents, with one out of every 27 isolates resistant to all tested antibiotics. The detection of high-risk clones and novel sequence types in hospital settings highlights the potential challenges to existing infections and prevention and control programs and treatment guidelines, further emphasizing the need for continuous surveillance and tailored infection control strategies to mitigate the spread of these drug-resistant pathogens in this low-resource setting (23).

Our study revealed a 14.57% prevalence of *P. aeruginosa* among patients with SSIs. This aligns with findings from similar studies in various countries, such as India, Ghana, Greece, and Saudi Arabia, which reported prevalence rates ranging from 7.9% to 20.1% (24–26). The comparable resistance rates highlight the global notoriety of the pathogen across countries.

Resistance to ciprofloxacin was found in 27% of the *P. aeruginosa* isolates, potentially due to its frequent usage in clinical settings driven by its lower cost and over-the-counter availability. Fluoroquinolone resistance rates vary across the African continent, from 6.9% to 61.1%, and highlight that resistance to the antimicrobial extends beyond Ghana (27–29). Increasing resistance to carbapenems, the last line of defense against *P. aeruginosa* and other Gram-negative infections, is a growing global concern in public health (30, 31). The finding of 12.2% carbapenem resistance in the *P. aeruginosa* isolates is particularly alarming, as resistance to this drug may result in treatment failure or mortality and further complicate the effective treatment of SSIs in Ghana.

We identified 50 different sequence types with global high-risk clones: ST235, ST308, and ST773, among others, in our collection, reflecting the genetic diversity of *P. aeruginosa* (12, 32, 33). Novel STs—ST4287, ST4288, ST4289, ST4521, ST4522, ST4523, and ST4524—were identified. The occurrence of multiple genotypes—globally dominating as well as novel clones—within the two hospitals indicates frequent recombination events in *P. aeruginosa*, supporting non-clonal structure reports of *P. aeruginosa* (33). The presence of newly identified STs suggests the possibility of unique evolutionary pressures in Ghana, warranting the need for further investigations and underscoring the need for continuous genomic surveillance to detect real-time emerging threats. Again, these newly identified sequence types could indicate an evolving genomic epidemiology of *P. aeruginosa* within the two hospitals.

An examination of the *P. aeruginosa* PubMLST database revealed a total of 3,621 global genomes, with 113 of these genomes originating from the African continent (accessed 8 July 2025). Within this data set, 76 international high-risk clones (ST233, ST235, ST244, ST277, ST357, ST308, ST446, ST654, and ST773) were identified, showing significant dispersion of these clones across West Africa and the broader continent (34). This contrasts with previous reports that identified ST111, ST233, ST235, and ST244 as the dominant high-risk strains on the continent (12, 35). This suggests the potential introduction of other high-risk clones onto the continent over time. SNP-level relatedness indicates possible transmission events or persistent colonization within the same hospital departments similar to what has been observed elsewhere (36). The findings point to intra-departmental spread of clones and underscore the need for targeted infection prevention and control measures in the affected clinical units.

Our analysis revealed a close association between specific *bla*_PDC_ alleles and certain *P. aeruginosa* sequence types. Similar to a report by Ozoaduche et al. (37), the ancestral *bla*_*PDC-1*_ was consistently present in all four strains of ST244, while *bla*_*PDC-16*_, *bla*_*PDC-19a*_, *bla*_*PDC-3*_, and *bla*_*PDC-5*_ were found in ST773, ST308, ST654, and ST277, respectively (37). Similar reports of *bla*_*PDC-3*_ and *bla*_*PDC-5*_, being the globally dominant PDC variants, were observed in our collection, although a sub-Saharan Africa regional prevalence of these variants was not determined (38). Likewise, the presence of *bla*_*PDC-16*_ in ST773 aligns with its noted prevalence in the high-risk clone (39). These findings suggest that *bla*_*PDC*_ variants may be conserved within particular STs, possibly due to vertical inheritance clonally, and may have implications for potential phenotypic cephalosporin resistance under adverse environmental pressures.

Despite the global prevalence of ST111 and ST235 high-risk clones reported, our study revealed a greater occurrence of ST308 and ST773 among the strains analyzed, suggesting a shift in the epidemiology of the organism in this region (12, 35). The detection of international high-risk clones—ST235, ST446, ST654, and ST773—with MDR/PDR phenotypes, harboring carbapenemases and of serotype O11, is consistent with earlier reports elsewhere (12, 40–42). The finding of MDR and high-risk clones with carbapenemase genes suggests that current treatment regimens may be inadequate for managing SSIs caused by *P. aeruginosa* in Ghana. This highlights the urgent need for updated treatment guidelines and IPC strategies within the two hospitals.

Three isolates showed resistance to all antipseudomonal agents tested: β-lactams, aminoglycosides, and fluoroquinolones. Of note, these isolates belong to high-risk international clones ST235 (*n* = 1) and ST773 (*n* = 2), both of which are globally disseminated and associated with enhanced antimicrobial resistance, virulence, and outbreak potential (12, 32, 41, 43). Their resistance phenotypes were supported by the presence of carbapenemase genes such as *bla_GES-1_* for ST235 and *bla_NDM-1_* for ST773, as well as multiple resistance determinants. Clinically pan drug resistant or extensively drug resistant *P. aeruginosa* infections have been associated with high mortality, prolonged hospitalization, and increased healthcare costs, especially when treatment options are limited (30, 31, 41). In resource-limited settings like Ghana, where access to last-line antimicrobials (e.g., cefiderocol, ceftolozane-tazobactam, imipenem-relebactam) is restricted or nonexistent, the occurrence of such clones severely limits therapeutic options and may lead to treatment failure (23, 41, 42). Also, persistence and hospital-wide transmission of these strains in surgical and intensive care units further complicate the challenge and underscore the need for genomic surveillance, enhanced IPC, and antimicrobial stewardship programs to prevent spread within the hospital setting (9, 11, 12, 40, 41, 44).

Our data also revealed global, endemic, and nosocomial strains whose presence has been reported in both human and non-human samples. Notable examples are ST277 and ST308, which were reported in clinical and environmental samples in Brazil and Singapore, respectively (14, 44). Unlike these studies, our ST277 and ST308 strains did not have any metallo-beta-lactamase (MBL) genes. This may be due to the regional differences in antimicrobial use and selective pressures in these two regions. Again, the observed phenotypic resistance in our strains may be through non-MBL means, such as multidrug efflux systems or mutations in target sites (45). This highlights the high adaptability of *P. aeruginosa* in different antimicrobial environments as well as the need for genomic surveillance using the One Health approach, as these strains circulate in human, animal, and environmental sources.

The enormous virulence potential of *P. aeruginosa* is evident in this study, with over 200 genes associated with toxin production, biofilm formation, immune evasion, and stress adaptation. Like earlier reports, *exoT* and *exoY* were abundantly dispersed compared with the mutually exclusive T3SS effectors *exoS* and *exoU* (46, 47). Similarly, *exoS*+ genotypes were the dominant genotypes compared with *exoU*+ strains, whose 21.95% prevalence is consistent with the 22.8% reported in earlier studies, as well as a co-presence of the two mutually exclusive genes (47, 48). These virulence factors influence pathogenicity and have severe implications for patient outcomes.

Unlike earlier reports by Horna et al. (47) and Sawa et al. (43), where the *exoU* genotype is associated with fluoroquinolone resistance, we observed fluoroquinolone resistance across different T3SS pathotypes, including *exoU+/exoS*−, *exoU−/exoS+*, and *exoU−/exoS−*, suggesting that genetic evolution may influence resistance over time (43, 47). Additionally, identifying *exoU*−/*exoS*− pathotype in one strain each of ST1203, ST1978, ST3453, and ST446 further underscores the genetic diversity within the isolates and suggests potential for new virulence combinations.

### Conclusion

This study provides insights into the genomic epidemiology of *P. aeruginosa* in a resource-limited setting. This useful knowledge, particularly with the occurrence of highly virulent and resistant clones, O-antigen serotypes, and novel sequence types, especially in our settings, is vital for the implementation of effective infection control measures and the development of treatment strategies in this low-resource setting.

These findings call for strengthened antimicrobial stewardship and genomic surveillance programs to monitor resistance trends and mitigate the spread of drug-resistant *P. aeruginosa* in Ghanaian healthcare settings.

## Data Availability

The data set is publicly available in the NCBI database under the BioProject number PRJNA1015653.
